# Primary Mediastinal Malignant Melanoma Presenting With Pleural Effusion: A Case Report

**DOI:** 10.1002/cnr2.70484

**Published:** 2026-02-02

**Authors:** Minlian Nong, Weifeng Wei, Naijian Li, Yunxiang Zeng, Jinlin Wang

**Affiliations:** ^1^ Department of Geriatrics Respiratory Medicine The First Affiliated Hospital of Guangxi Medical University Nanning People's Republic of China; ^2^ Department of Pulmonary and Critical Care Medicine, State Key Laboratory of Respiratory Disease, National Center for Respiratory Medicine, Guangzhou Institute of Respiratory Health, The First Affiliated Hospital Guangzhou Medical University Guangzhou People's Republic of China

## Abstract

**Background:**

Primary mediastinal malignant melanoma (PMMM) presenting with pleural effusion as the initial manifestation is exceedingly rare, presenting significant diagnostic and therapeutic challenges. This article reports a case of PMMM that initially manifested as pleural effusion and includes a review of the relevant literature.

**Case:**

The patient was admitted with pleural effusion and an anterior mediastinal mass. A pleural biopsy and fine‐needle aspiration of the mediastinal mass led to a pathological diagnosis of malignant melanoma. Ultimately, based on clinical findings, a final diagnosis of PMMM was established. The patient subsequently developed pleural hemorrhage; although hemostasis was achieved through thoracoscopy, his condition stabilized only temporarily. He ultimately succumbed to lung infection and multiple organ failure.

**Conclusion:**

PMMM presenting with pleural effusion lacks distinct clinical features, highlighting the importance of pathological diagnosis. Pleural hemorrhage associated with pleural metastasis may be a characteristic feature, and the prognosis for such cases is poor. Enhanced recognition and early intervention are essential to improve outcomes.

## 1 Introduction

Malignant melanoma (MM) is a highly aggressive tumor that originates from neural crest melanocytes, characterized by early metastasis, strong invasiveness, and a poor prognosis. MM predominantly occurs in the skin (91.2%), followed by the eyes (5.3%), mucous membranes (1.3%), and other sites (2.2%) [1, 2]. Primary mediastinal malignant melanoma (PMMM) is extremely rare, with only a limited number of cases reported to date [3, 4, 5, 6, 7]. Its initial presentation can range from a localized lesion to rapidly invasive disease affecting multiple organ systems, and detailed descriptions of its clinical characteristics are scarce, complicating the diagnostic approach. While pleural effusion due to thoracic metastasis can occur in cutaneous malignant melanoma [8] and PMMM, diagnosing and treating patients who present with pleural effusion is particularly challenging. This paper reports a rare case of PMMM with pleural metastasis, analyzing its clinical presentation, imaging features, diagnostic criteria, and therapeutic outcomes to enhance understanding of this disease.

## 2 Case Presentation

**FIGURE 1:**
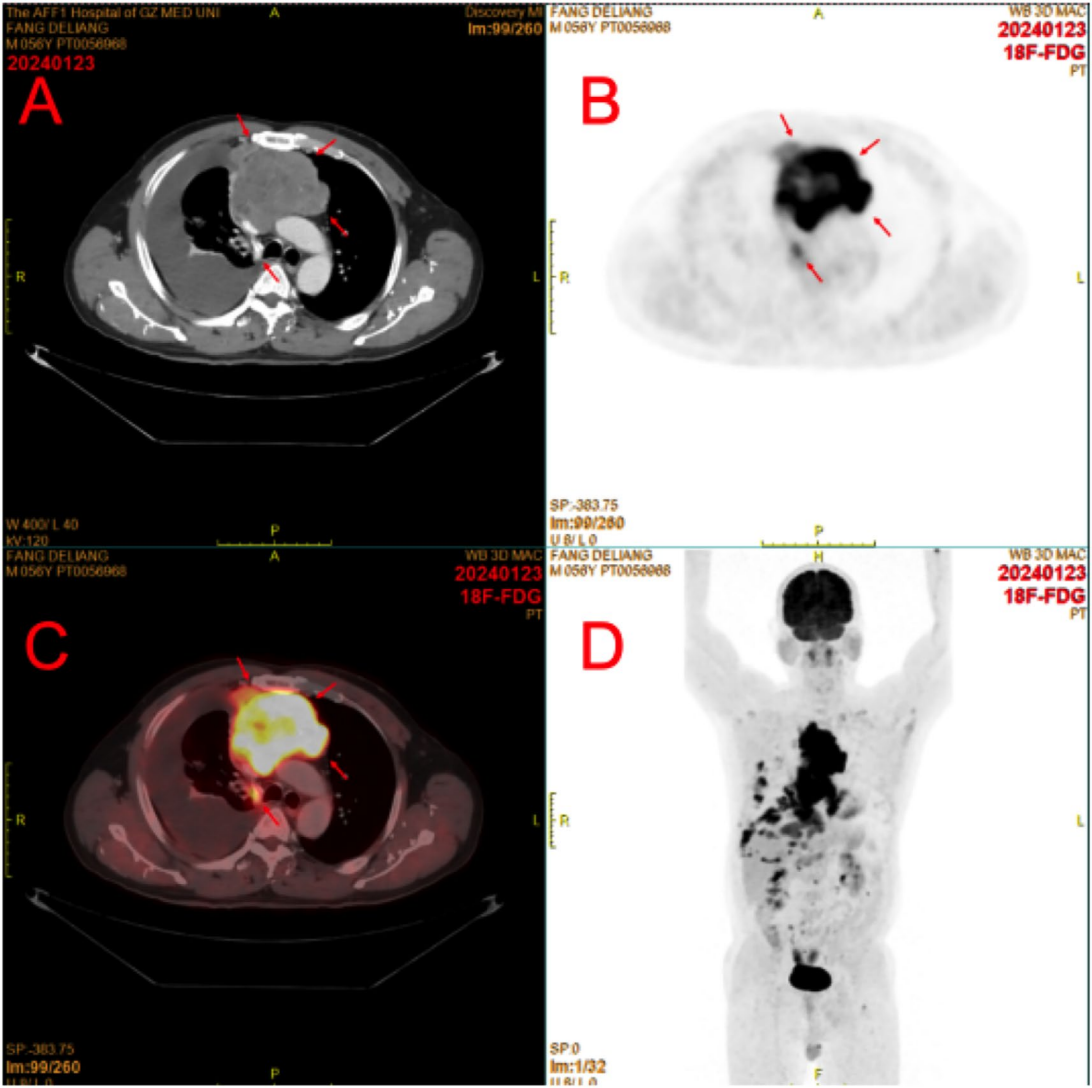
PET‐CT imaging results of the patient with primary malignant melanoma of the mediastinum. (A–C) High metabolic tumor lesions in the anterior mediastinum, with CT enhancement scan showing mild heterogeneous enhancement. Extensive tumor metastasis of the right pleura, accompanied by a large amount of pleural effusion on the right side. (D) Multiple tumor metastases in the right pulmonary hilum, hepatogastric space, around the abdominal aorta, and around the mesentery.

**FIGURE 2:**
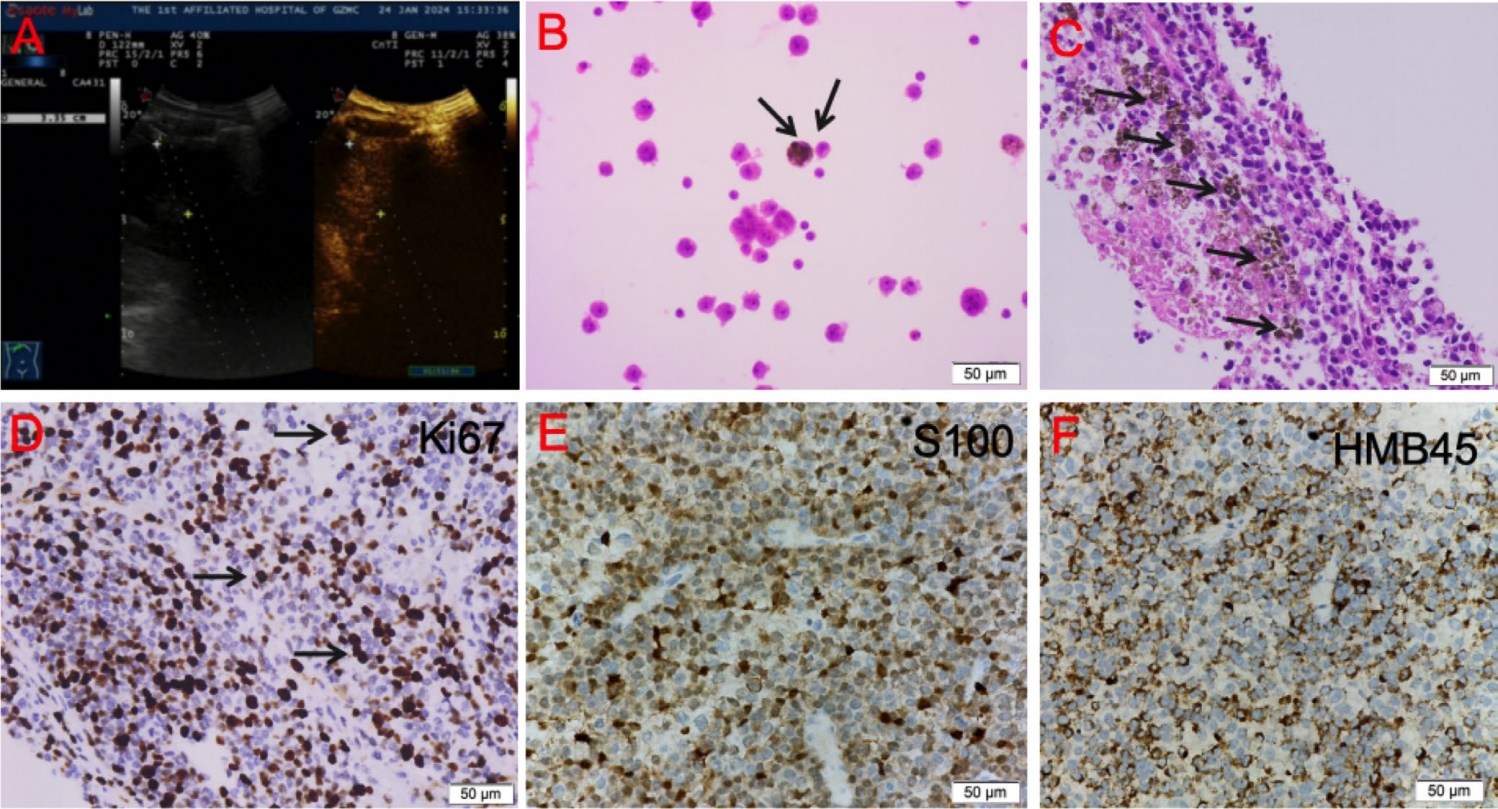
Ultrasound‐guided mediastinal tumor biopsy. (A) Ultrasound‐guided mediastinal tumor biopsy. (B) Pathology of pleural effusion sediment showing typical melanoma cells. (C) HE staining of the mediastinal tumor biopsy specimen under high magnification showing sheets of tumor cells with round or oval nuclei, deeply stained nuclei, sparse cytoplasm, focal necrosis, and pigment deposition. (D–F) The pathology shows a positive melanoma marker.

A 57‐year‐old male patient was admitted to our hospital (The First Affiliated Hospital of Guangzhou Medical University) in December 2023, presenting with a 1‐week history of cough and shortness of breath. He had no prior history of malignancy. Enhanced chest CT and PET‐CT performed at admission revealed the following findings: (1) a large, lobulated, hypermetabolic mass in the anterior mediastinum, measuring approximately 6.7 × 7.5 × 13.5 cm, with heterogeneous density and no obvious calcification or fat density (Figure 1A,B); (2) involvement of the pericardium, along with compression and narrowing of the left and right brachiocephalic veins and the superior vena cava (Figure 1C); and (3) extensive right‐sided pleural metastasis, associated with massive right‐sided pleural effusion and multiple small nodules in both lungs, suggestive of possible metastatic tumors (Figure 1D).

Laboratory tests indicated a white blood cell count of 15.84 × 10^9^/L, with 64.1% neutrophils, and a hemoglobin level of 144 g/L. Lung tumor markers were elevated, with NSE at 27.80 ng/mL and CA125 at 60.50 U/mL; however, CEA, CA153, SCC, and CYFRA21‐1 levels were within normal ranges. Cardiac, liver, and renal functions, coagulation profile, infection markers, as well as routine urine and stool tests were normal.

Upon admission, the patient underwent thoracentesis and a pleural biopsy on the right side using standard pleural biopsy techniques. Following the PET/CT results, a real‐time ultrasound‐guided fine‐needle aspiration biopsy of the mediastinal mass was performed (Figure 2A). The pleural fluid collected was red, turbid, and free of clots. Laboratory tests indicated a positive Rivolta test (+) and a strongly positive Pandy test (+++). Analysis of the pleural fluid revealed elevated white blood cell counts at 3167 × 10^6^/L and red blood cell counts at 276 000 × 10^6^/L, with a differential showing 95% segmented neutrophils and 5% mononuclear cells. Large cells were observed at a rate of 3–5 per high power field (HPF). Biochemical analysis indicated total protein levels of 37.7 g/L, LDH levels of 1889 U/L, and ADA levels of 20.50 U/L. Tumor markers in the pleural fluid included NSE at 79.10 ng/mL, CEA at 1.63 ng/mL, and CA125 at 746.0 U/mL. The glucose level was measured at 5.41 mmol/L, with eosinophils accounting for 0.5%. Tests for TB‐DNA, tuberculosis smear, and bacterial/fungal cultures all returned negative results.

Cytological examination of the pleural fluid sediment revealed numerous cells with thickened nuclear membranes and prominent nucleoli, some containing pigments, indicative of malignant tumor cells (Figure 2B). Pathological analysis of the parietal pleura and mediastinal mass demonstrated fragments of tissue exhibiting sheet‐like distributions of tumor cells. Immunohistochemical results were as follows: CK (−), HMB45 (+), S‐100 (+), Ki67 approximately 50% positive, ERG positive in blood vessels, and SALL4 (−). These findings were consistent with a diagnosis of malignant melanoma (Figure 2C–F). The cells displayed round or oval nuclei with deep staining and sparse cytoplasm, along with focal necrosis and pigment deposition. Comprehensive head MRI revealed no evidence of primary or metastatic lesions (images not shown).

Five days after the pleural biopsy, the patient experienced worsening dyspnea and progressive anemia, with hemoglobin levels dropping from 120 to 62 g/L, alongside an increase in right‐sided pleural effusion. Suspecting active intrathoracic bleeding, an exploratory thoracoscopy and hemostasis were performed under general anesthesia. On the seventh postoperative day, the patient developed a high fever (reaching up to 39°C) and worsening respiratory distress, characterized by a rapid heart rate and widespread wheezing and crackles in both lungs. The following day, the patient's condition deteriorated further, with a drop in blood pressure, exacerbated dyspnea, and persistent high fever. Laboratory tests indicated progressively elevated inflammatory markers and stable hemoglobin levels, leading to a diagnosis of septic shock accompanied by acute kidney injury, hyperkalemia, and metabolic acidosis. Despite intensive treatment, the patient ultimately succumbed to the illness.

## 3 Discussion

Primary mediastinal malignant melanoma (PMMM) is an extraordinarily rare entity, with fewer than 20 cases reported in the literature. Its diagnosis requires rigorous exclusion of primary melanocytic lesions in other sites, and its pathogenesis remains speculative. Our case highlights the diagnostic challenges and aggressive nature of PMMM, particularly when presenting with pleural effusion as the initial manifestation.

The management of PMMM remains poorly defined due to its rarity. Systemic therapies for advanced melanoma, including immune checkpoint inhibitors (e.g., ipilimumab/nivolumab, pembrolizumab) and BRAF/MEK inhibitors, have revolutionized outcomes in cutaneous melanoma [9, 10, 11]. While data specific to PMMM are limited, these agents may hold promise. For instance, nivolumab combined with ipilimumab has shown durable responses in mucosal melanomas, and BRAF inhibitors (e.g., dabrafenib) could benefit patients with BRAF V600E mutations. In our case, the patient's rapid clinical deterioration precluded targeted therapy initiation, underscoring the need for early molecular profiling in such scenarios. Whilst the use of intrapleural bevacizumab has not been directly assessed in melanoma, evidence supports its efficacy in reducing malignant pleural effusions in non‐small cell lung cancer [12]. Further exploration for PMMM‐related effusions is warranted.

The origin of PMMM is debated. Melanocytes are derived from neural crest cells during embryogenesis, and ectopic migration may explain primary visceral melanomas. For instance, primary esophageal or pulmonary melanomas are hypothesized to arise from residual melanoblasts in mucosal or mesenchymal tissues [13]. Similarly, the mediastinum may harbor such ectopic cells. The classification of our case as PMMM rather than “metastatic melanoma of unknown primary” hinges on two criteria: (1) the mediastinal mass as the dominant lesion with metastatic spread to adjacent structures (pleura, pericardium), and (2) exhaustive exclusion of alternative primary sites (including dermatological, ophthalmological, endoscopic exams and PET‐CT exams). Given the rarity of PMMM, prognosis remains poorly characterized but appears worse than cutaneous melanoma, aligning with the aggressive nature of mucosal melanomas [14]. While we classified this case as PMMM per published criteria, the possibility of an occult primary melanoma cannot be entirely excluded [3, 4, 5, 6, 7]. Molecular profiling (e.g., comparative genomic analysis of mediastinal and pleural lesions) could help clarify clonal origins in future cases. Table 1 summarizes published PMMM cases, revealing heterogeneity in presentation and outcomes [3, 4, 5].

**TABLE 1:** Clinical summary of published primary mediastinal malignant melanoma cases.

Study (year)	Age/sex	Presentation	Management	Outcome
Karuppiah (2006) [3]	62/M	Mediastinal mass	Biopsy	Died (6 months)
Park (2012) [4]	45/M	Anterior mediastinal mass	Biopsy	Unknown
Park (2012) [4]	41/F	Anterior mediastinal mass	Biopsy + Chemo	Died (4 months)
Li (2017) [5]	58/M	Pleural effusion	Chemo	Died (3 months)
Current case	57/M	Pleural effusion	Biopsy + supportive care	Died (1 month)

Upon admission and based on the CT results, relevant examinations were conducted, with thoracentesis and pleural biopsy identified as critical first steps in the diagnosis and treatment of pleural effusion. Specific indicators in the pleural fluid, such as CEA, ADA, and bacteriological tests, did not yield valuable diagnostic information. Literature reports indicate that black pleural effusion is a unique presentation of malignant melanoma, attributed to the presence of melanocytes in the effusion [15]; however, this was not observed in our patient. Due to its extremely low incidence and nonspecific clinical features, cytodiagnosis of malignant melanoma is often challenging, particularly in cases of amelanotic melanoma [16]. While medical thoracoscopy is typically employed to obtain sufficient pleural tissue for diagnosis [15], it can be more invasive for patients. In our case, we utilized minimally invasive standard pleural biopsy for the pleural biopsy [17]. Fortunately, both cytological analysis of the pleural fluid and the results from the pleural biopsy confirmed that the malignant pleural effusion was caused by malignant melanoma. Therefore, it is essential to emphasize the correct diagnostic procedures for the diagnosis of pleural malignant melanoma or pleural metastasis from malignant melanoma.

Pleural hemorrhage, as observed in our patient, may be a hallmark of PMMM with pleural metastasis. Elevated LDH levels (1889 U/L) and hemorrhagic effusion likely reflect tumor aggressiveness and vascular invasion. Notably, LDH is a recognized prognostic marker in malignant pleural effusions, and its extreme elevation here correlates with the patient's rapid decline. Thoracoscopic hemostasis provided transient stabilization, but subsequent sepsis and multi‐organ failure highlight the vulnerability of these patients to iatrogenic complications.

In conclusion, PMMM presenting with pleural effusion lacks distinct clinical features, highlighting the importance of pathological diagnosis. Pleural hemorrhage associated with pleural metastasis may be a characteristic feature. While the prognosis of PMMM remains poor, particularly with pleural involvement, emerging immunotherapies (e.g., ipilimumab/nivolumab) have significantly improved outcomes in advanced melanoma, underscoring the need for early molecular profiling and multidisciplinary intervention.

## Author Contributions

**Minlian Nong** and **Weifeng Wei:** conception and design of the study, data collection, and drafting of the manuscript. **Naijian Li** and **Yunxiang Zeng:** the data acquisition and interpretation, as well as literature review. **Jinlin Wang:** conceptualization, writing – review and editing, funding acquisition, project administration.

## Funding

This work was supported by the National Major Science and Technology Project for Prevention and Treatment of Cancer, Cardiovascular and Cerebrovascular Diseases, Respiratory Diseases, and Metabolic Diseases (Grant No. 2024ZD0522803/2024ZD0522800), the Natural Science Foundation of China (Grant No. 82570134), the Natural Science Foundation of Guangdong Province (Grant No. 2025A1515012002), and the Science and Technology Innovation Committee Joint Funding of Guangzhou (No. 2023A03J0356).

## Consent

This study is a case report and literature review; consent has been obtained from the patient's family.

## Conflicts of Interest

The authors declare no conflicts of interest.

## Data Availability

The data that support the findings of this study are available on request from the corresponding author. The data are not publicly available due to privacy or ethical restrictions.
